# Molecular testing and treatment patterns for patients with advanced non-small cell lung cancer: PIvOTAL observational study

**DOI:** 10.1371/journal.pone.0202865

**Published:** 2018-08-27

**Authors:** Dae Ho Lee, Ming-Sound Tsao, Karl-Otto Kambartel, Hiroshi Isobe, Ming-Shyan Huang, Carlos H. Barrios, Adnan Khattak, Filippo de Marinis, Smita Kothari, Ashwini Arunachalam, Xiting Cao, Thomas Burke, Amparo Valladares, Javier de Castro

**Affiliations:** 1 Asan Medical Center, Seoul, Republic of Korea; 2 University Health Network, Princess Margaret Cancer Centre, Toronto, Canada; 3 Krankenhaus Bethanien, Moers, Germany; 4 KKR Sapporo Medical Center, Sapporo-shi, Hokkaido, Japan; 5 Kaohsiung Medical University Hospital, College of Medicine, Kaohsiung Medical University, Taiwan; 6 Hospital do Câncer Mãe de Deus, Porto Alegre, Brazil; 7 Fiona Stanley Hospital, Murdoch, Western Australia, Australia; 8 European Institute of Oncology, IRCCS, Milan, Italy; 9 Center for Observational and Real World Evidence (CORE), Merck & Co., Inc., Kenilworth, NJ, United States of America; 10 Outcomes Research, Merck Sharp & Dohme de España, Madrid, Spain; 11 Hospital Universitario La Paz (IDIPAZ), Madrid, Spain; University of South Alabama Mitchell Cancer Institute, UNITED STATES

## Abstract

**Background:**

The goals of this multinational retrospective study were to describe treatment patterns and survival outcomes by receipt of molecular testing and molecular status of patients with advanced non-small cell lung cancer (NSCLC).

**Methods:**

This chart review study, conducted in Italy, Spain, Germany, Australia, Japan, Korea, Taiwan, and Brazil, included 1440 patients with newly diagnosed advanced (stage IIIB/IV) NSCLC initiating systemic therapy from January 2011 through June 2013, with follow-up until July 2016. We evaluated treatment patterns and survival by histology, line of therapy, molecular testing, and test results for epidermal growth factor receptor (*EGFR*) mutation and/or anaplastic lymphoma kinase (*ALK*) rearrangement. Country-specific data were analyzed descriptively and presented as ranges (lowest to highest country). Overall survival (OS) was estimated using Kaplan-Meier method.

**Results:**

Patients with ≥1 molecular test varied from 43% (Brazil) to 85% (Taiwan). Numerically greater proportions of patients who were female, Asian, or never/former-smokers, and those with nonsquamous histology or stage-IV NSCLC, received a test. Testing was common for nonsquamous NSCLC (54%, Brazil, to 91%, Taiwan), with positive *EGFR* and *ALK* tests from 17% (Brazil and Spain) to 67% (Taiwan) and from 0% (Brazil) to 60% (Taiwan), respectively. First-line treatment regimens for nonsquamous NSCLC with positive *EGFR/ALK* tests included targeted therapy for 30% (Germany) to 89% (Japan); with negative/inconclusive test results, platinum-based combinations for 88% (Japan) to 98% (Brazil); and if not tested, platinum-based combinations for 80% (Australia) to 95% (Japan), except in Taiwan, where 44% received single agents. Median OS from first-line therapy initiation was 10.0 (Japan) to 26.7 (Taiwan) months for those tested and 7.6 (Australia/Brazil) to 19.3 (Taiwan) months for those not tested.

**Conclusions:**

We observed substantial variation among countries in testing percentages, treatment patterns, and survival outcomes. Efforts to optimize molecular testing rates should be implemented in the context of each country’s health care scenario.

## Introduction

Lung cancer is the leading type of cancer and cause of cancer-related death worldwide [1–3]. Non-small cell lung cancer (NSCLC) comprises >80% of histologically confirmed lung cancers. Amongst NSCLC histologies, squamous cell carcinoma and adenocarcinoma are the most common, comprising about 30% and 50%, respectively [4, 5]. The 5-year survival rates are low for advanced NSCLC: 5% for stage IIIB and 1% for stage IV [4].

Recent years have seen an ever-expanding role of more precise histological diagnosis and molecular testing in guiding treatment decisions for patients with NSCLC [6, 7]. Agents that target specific molecules or cell signaling pathways, such as the epidermal growth factor receptor (EGFR) tyrosine kinase inhibitors (TKIs) and anaplastic lymphoma kinase (ALK) inhibitors, are standard now for treating advanced NSCLC [5, 8]. National and international NSCLC clinical guidelines recommend that patients with advanced NSCLC testing positive for *EGFR* mutation or *ALK* rearrangement be treated with an EGFR TKI or ALK inhibitor, respectively, for first-line therapy or, alternatively, for sequential first-line or second-line therapy if mutations are discovered during the course of first-line treatment [5, 8, 9]. First-line therapy with an EGFR TKI (eg, erlotinib, gefitinib, afatinib) significantly prolongs progression-free survival (PFS) and is associated with significantly higher tumor response rate when compared with first-line cytotoxic chemotherapy for patients with *EGFR* mutations [10]. Similar results were reported in recent studies of ALK inhibitor therapies (e.g., crizotinib, ceritinib, alectinib) for patients with *ALK*-rearranged NSCLC [11].

Both *EGFR* mutations and *ALK* rearrangements, while usually mutually exclusive, are found most commonly in lung adenocarcinoma and in non-smokers or never-smokers. In addition, *EGFR* mutations are found most commonly in women and patients of Asian descent, while *ALK* rearrangements are more common in younger patients [5, 12–16]. Recent guideline recommendations are that all patients with advanced-stage nonsquamous NSCLC should be tested for both *EGFR* mutations and *ALK* rearrangements before initiation of first-line treatment [5, 8, 17, 18]. For patients with squamous cell carcinoma, testing for both *EGFR* mutations and *ALK* rearrangements is recommended for never smokers and if the biopsy specimens are of mixed histology or are small and cannot exclude an adenocarcinoma component. Some consensus statements and local policies advocate reflex molecular testing upon diagnosis of nonsquamous NSCLC, regardless of clinical stage [19, 20].

In real-world clinical practice, the uptake of testing and treatment recommendations depends on many factors, including local guidelines, drug approval timelines, and reimbursement policies. Many, if not most, NSCLC diagnoses are made on the basis of small biopsies or cytology samples, and procuring sufficient tissue material for both histological subtyping of NSCLC, as well as molecular testing, is an important goal to guide individualized treatment decisions [18, 21–24]. The results of several observational (non-interventional) studies of NSCLC treatment patterns conducted in recent years leading up to 2013 indicate that testing for *EGFR* mutation is increasing over time in conjunction with increased prescribing of EGFR TKIs [25–31]. Observational data provide important information regarding practice patterns and treatment outcomes in the real-world clinical setting; this information complements the findings of randomized controlled trials, and can be used by policymakers and health care providers to assess and improve current care. However, there is globally a lack of observational data on practice patterns for advanced NSCLC including molecular testing and all treatment regimens [32].

The goals of this multinational retrospective study were to descriptively look at demographics, clinical characteristics, treatment patterns, and survival outcomes, by receipt of molecular testing and molecular status, for patients with advanced NSCLC who received first-line systemic therapy in the real-world practice setting. Our focus was on molecular testing for two predictive biomarkers, *EGFR* mutation and *ALK* rearrangement, for which targeted therapies were approved and available during the study years. The overall treatment pattern and health care resource use data from the study have been published [33–35].

## Methods

### Study design and patients

This was a non-interventional multinational retrospective study drawing on de-identified patient data abstracted from medical records of patients who received therapy for advanced NSCLC at academic and community sites in eight countries (Italy, Spain, Germany, Australia, Japan, Korea, Taiwan and Brazil). Detailed study methods have been previously published [33].

Approximately 150–200 adult patients (≥18 years of age) per country who initiated first-line therapy for a histologically and/or cytologically confirmed new diagnosis of stage IIIB or stage IV NSCLC during the eligibility period from January 1, 2011, to July 1, 2013 (to July 1, 2014, in Germany) were included in the study. Patients who did not receive systemic therapy, who were participating in a clinical trial, and who had an initial diagnosis of stage I, II, or IIIA NSCLC were excluded from the study. All patients who met the eligibility criteria were selected by medical record review, working backwards in time from the end of the eligibility period (July 1, 2013) until a suitable number of patients was reached for each center. Patients’ data were included from the date of initiating first-line therapy until the end of the study follow-up period, loss to follow-up, or death, whichever was first. The study follow-up period ended on the data abstraction date for each country, ranging from April 24, 2015, in Australia to May 27, 2016, in Brazil [33].

The study protocol was approved by the appropriate institutional review board or independent ethics committee for each study site. Informed consent was collected for patients from Italy, Spain, Germany, and Brazil who were alive at the time of data abstraction; an informed consent form was not required from deceased patients’ next of kin. In the other countries, informed consent was not required for working with the de-identified retrospective data used in the study. Full details of ethical approvals have been published [35] and the names of the institutional review boards / independent ethics committees for each study site are provided in Supporting S1 Table. All data in the patient records used in this retrospective study were fully deidentified / anonymized before any of the study authors accessed them.

### Data collection

Data on baseline patient demographic and clinical characteristics, NSCLC histology, treatment patterns, and biopsy and molecular testing practices were abstracted from medical records using electronic case report forms.

We collected information throughout the study period for each patient on types of molecular tests, frequency of tests, timing relative to diagnosis and start of therapy, type of tissue used for testing (archival or new), test turnaround time, and the health care personnel involved in ordering these tests. Results of testing for the two predictive biomarkers *EGFR* mutation and *ALK* rearrangement were collected. Treatment patterns and outcomes were collected for all patients and presented by histology, receipt of biomarker test, and mutation status. In addition, we collected the following biopsy-related variables: number of biopsies, timing of biopsy relative to diagnosis and start of therapy, site (primary or metastatic), size of tissue, method of biopsy, reason for biopsy, rebiopsy rate, timing of rebiopsy, and reason for rebiopsy. Study definitions of these variables are presented in the S1 File.

### Statistical analysis

Data were analyzed descriptively and reported using summary statistics for each country. We summarized patient and clinical characteristics by receipt of a molecular test at any time during the study (yes/no). Biopsy and biomarker practice patterns were presented at the patient level and at a biopsy or test level, respectively. Treatment patterns and survival were stratified and reported by receipt of predictive biomarker test (EGFR/ALK; yes/no), by mutation status amongst those who received a test (*EGFR/ALK*-positive or *EGFR/ALK*-negative/unknown), and by histology (squamous, nonsquamous, and all patients).

Overall survival (OS) was estimated for each country from the start of first-line and second-line therapy until the end of follow-up using the Kaplan-Meier product-limit method. Patients alive at the end of follow-up were censored on the date of last contact in the OS analyses.

This was an observational study with no *a priori* hypothesis testing; therefore, we did not undertake a formal calculation of sample size and statistical power. We reported the proportion of available data for key variables; missing data were not imputed. All analyses were carried out using SAS versions 9.2, 9.3, and 9.4 (SAS Institute, Cary, NC, USA).

## Results

### Patients

A total of 1440 patients were included in the study from 78 academic and community oncology sites in 8 countries. The demographic and clinical characteristics of all patients have been described by country in previous publications [33, 34]. The majority of patients in each country were male (from 53% in Germany to 77% in Spain) and current or former smokers (63% in Korea to 86% in Australia), with the exception of Taiwan (47% male and 33% current or former smokers). The median age in each country ranged from 63 to 70 years. Approximately three-quarters of patients had nonsquamous histology (including 93% in Taiwan), and over 77% in each country presented with stage IV disease.

### Molecular testing patterns

Tables 1 and 2 summarize the characteristics of patients in each country by whether they received at least one molecular test of any kind during the study period. Three-quarters or more of all patients received one or more molecular tests in Spain (76%), Japan (74%), Korea (76%), and Taiwan (85%); testing rates in the other countries ranged from 43% in Brazil to 61% in Australia.

**Table 1:** Demographic and clinical characteristics of patients by receipt of one or more molecular tests^a^ (yes/no) in Italy, Spain, Germany, and Australia.

	Italy		Spain		Germany		Australia	
	(N = 174)		(N = 202)		(N = 139)		(N = 208)	
Characteristic	Tested	Not tested	Tested	Not tested	Tested	Not tested	Tested	Not tested
N (%)	89 (51)	85 (49)	154 (76)	48 (24)	78 (56)	61 (44)	126 (61)	82 (39)
Histology, n (%)								
Squamous	1 (2)	41 (98)	14 (42)	19 (58)	6 (21)	22 (79)	3 (10)	27 (90)
Nonsquamous	79 (65)	42 (35)	119 (85)	21 (15)	71 (66)	37 (34)	115 (71)	46 (29)
Unknown	9 (82)	2 (18)	21 (72)	8 (28)	1 (33)	2 (67)	8 (47)	9 (53)
Sex, n (%)								
Male	50 (41)	73 (59)	116 (75)	39 (25)	33 (45)	41 (55)	65 (52)	60 (48)
Female	39 (77)	12 (24)	38 (81)	9 (19)	45 (69)	20 (31)	61 (74)	22 (27)
Age, n (%)								
<75 years	77 (55)	63 (45)	129 (77)	39 (23)	67 (58)	48 (42)	108 (60)	71 (40)
≥75 years	12 (35)	22 (65)	25 (74)	9 (27)	11 (46)	13 (54)	18 (62)	11 (38)
Age, years								
Mean (SD)	63 (11)	66 (10)	63 (10)	63 (11)	62 (11)	65 (10)	64 (10)	63 (10)
Median (range)	64 (28–86)	67 (38–84)	64 (41–84)	61 (40–84)	62 (33–81)	66 (42–81)	63 (37–89)	65 (39–82)
Race, n (%)^b^								
White	89 (51)	84 (49)	151 (76)	48 (24)	78 (56)	62 (44)	93 (62)	56 (38)
Asian	0	0	0	0	0	0	11 (69)	5 (31)
Black	0	0	2 (100)	0	0	0	1 (100)	0
Other	0	0	0	0	0	0	7 (58)	5 (42)
Unknown	0	0	1 (100)	0	0	0	14 (47)	16 (53)
Smoking history, n (%)								
missing	0	0	0	0	2	1	0	0
Current	14 (38)	23 (62)	47 (70)	20 (30)	22 (46)	26 (54)	19 (35)	35 (65)
Former	33 (41)	48 (59)	82 (78)	23 (22)	26 (55)	21 (45)	82 (66)	43 (34)
Never	23 (70)	10 (30)	24 (86)	4 (14)	19 (83)	4 (17)	22 (88)	3 (12)
Unknown	19 (83)	4 (17)	1 (50)	1 (50)	9 (50)	9 (50)	3 (75)	1 (25)
Stage at diagnosis, n (%)								
IIIB	6 (46)	7 (54)	8 (47)	9 (53)	9 (29)	22 (71)	23 (53)	21 (48)
IV	83 (52)	78 (48)	146 (79)	39 (21)	69 (64)	39 (36)	103 (63)	61 (37)
ECOG PS, n (%)								
Missing	30	45	50	23	18	18	32	23
0	22 (63)	13 (37)	27 (84)	5 (16)	43 (64)	24 (36)	36 (66)	19 (35)
1	31 (60)	21 (40)	49 (83)	10 (17)	16 (50)	16 (50)	50 (60)	33 (40)
2	4 (40)	6 (60)	18 (75)	6 (25)	1 (33)	2 (67)	6 (55)	5 (46)
3	2 (100)	0	8 (67)	4 (33)	0	1 (100)	2 (67)	1 (33)
4	0	0	2 (100)	0	0	0	0	1 (100)
Line of therapy received, n (%)								
First-line	89 (51)	85 (49)	154 (76)	48 (24)	78 (56)	61 (44)	126 (61)	82 (39)
Second-line	56 (55)	45 (45)	79 (82)	17 (18)	43 (67)	21 (33)	88 (69)	40 (31)
Third-line	28 (64)	16 (36)	40 (87)	6 (13)	22 (71)	9 (29)	40 (76)	13 (25)

Note: All percentages are row percentages for each country.

ECOG PS, Eastern Cooperative Oncology Group performance status.

^a^Molecular tests could include those for epidermal growth factor receptor (*EGFR*) and/or *KRAS* mutation and/or anaplastic lymphoma kinase (*ALK*) rearrangement and/or other (not defined) molecular test.

^b^Race data missing for 1 patient in Italy who was not tested.

**Table 2:** Demographic and clinical characteristics of patients by receipt of one or more molecular tests^a^ (yes/no) in Japan, Korea, Taiwan, and Brazil.

	Japan		Korea		Taiwan		Brazil	
	(N = 175)		(N = 150)		(N = 217)		(N = 175)	
Characteristic	Tested	Not tested	Tested	Not tested	Tested	Not tested	Tested	Not tested
N (%)	130 (74)	45 (26)	114 (76)	36 (24)	185 (85)	32 (15)	75 (43)	100 (57)
Histology, n (%)								
Squamous	17 (40)	26 (61)	7 (23)	23 (77)	2 (13)	14 (88)	2 (6)	33 (94)
Nonsquamous	110 (85)	19 (15)	101 (89)	12 (11)	183 (91)	18 (9)	71 (54)	61 (46)
Unknown	3 (100)	0	6 (86)	1 (14)	0	0	2 (25)	6 (75)
Sex, n (%)								
Male	83 (68)	40 (33)	71 (68)	33 (32)	81 (79)	22 (21)	39 (34)	76 (66)
Female	47 (90)	5 (10)	43 (94)	3 (7)	104 (91)	10 (9)	36 (60)	24 (40)
Age, n (%)								
<75 years	88 (72)	35 (29)	104 (77)	32 (24)	146 (86)	24 (14)	66 (44)	84 (56)
≥75 years	42 (81)	10 (19)	10 (71)	4 (29)	39 (83)	8 (17)	9 (36)	16 (64)
Age, years								
Mean (SD)	69 (8)	68 (7)	61 (10)	64 (10)	65 (12)	63 (13)	61 (11)	65 (9)
Median (range)	70 (47–86)	69 (52–79)	64 (31–82)	66 (41–83)	66 (30–92)	64 (38–79)	61 (41–83)	66 (35–85)
Race, n (%)								
White	0	0	0	0	0	0	37 (48)	40 (52)
Asian	130 (74)	45 (26)	114 (76)	36 (24)	184 (85)	32 (15)	1 (100)	0
Black	0	0	0	0	1 (100)	0	2 (22)	7 (78)
Other	0	0	0	0	0	0	0	1 (100)
Unknown	0	0	0	0	0	0	35 (40)	52 (60)
Smoking history, n (%)								
Current	10 (48)	11 (52)	32 (63)	19 (37)	15 (75)	5 (25)	9 (35)	17 (65)
Former	87 (73)	33 (28)	32 (73)	12 (27)	40 (78)	11 (22)	45 (41)	65 (59)
Never	32 (97)	1 (3)	46 (90)	5 (10)	129 (90)	14 (10)	19 (54)	16 (46)
Unknown	1 (100)	0	4 (100)	0	1 (33)	2 (67)	2 (50)	2 (50)
Stage at diagnosis, n (%)								
IIIB	20 (69)	9 (31)	15 (58)	11 (42)	14 (70)	6 (30)	6 (33)	12 (67)
IV	110 (75)	36 (25)	99 (80)	25 (20)	171 (87)	26 (13)	69 (44)	88 (56)
ECOG PS, n (%)								
Missing	46	21	63	19	12	0	9	34
0	37 (79)	10 (21)	33 (92)	3 (8)	35 (76)	11 (24)	23 (72)	9 (28)
1	34 (77)	10 (23)	14 (56)	11 (44)	101 (89)	13 (11)	32 (50)	32 (50)
2	8 (80)	2 (20)	1 (25)	3 (75)	27 (77)	8 (23)	10 (30)	23 (70)
3	5 (71)	2 (29)	3 (100)	0	6 (100)	0	1 (33)	2 (67)
4	0	0	0	0	4 (100)	0	0	0
Line of therapy received, n (%)								
First-line	130 (74)	45 (26)	114 (76)	36 (24)	185 (85)	32 (15)	75 (43)	100 (57)
Second-line	78 (74)	27 (26)	75 (78)	21 (22)	129 (83)	26 (17)	51 (56)	40 (44)
Third-line	44 (80)	11 (20)	45 (76)	14 (24)	78 (86)	13 (14)	21 (70)	9 (30)

Note: All percentages are row percentages for each country.

ECOG PS, Eastern Cooperative Oncology Group performance status.

^a^Molecular tests could include those for epidermal growth factor receptor (*EGFR*) and/or *KRAS* mutation and/or anaplastic lymphoma kinase (*ALK*) rearrangement and/or other (not defined) molecular test.

A higher percentage of patients with nonsquamous histology had a molecular test than those with squamous histology. Moreover, numerically more women than men were tested, as were Asian vs. white or black patients, never smokers and ex-smokers vs. current smokers, and patients with stage IV vs. stage IIIB NSCLC (Tables 1 and 2). However, a relatively high proportion of male patients had a molecular test in Japan and Korea (68%), Spain (75%), and Taiwan (79%). Numerically greater proportion of patients ≥75 years old (vs. <75 years) had a molecular test in Japan, whereas lower proportions of patients ≥75 years old (vs. <75 years) in Italy and Germany had a test; in the other countries, there were minimal differences according to these two age groups in whether patients were tested (Tables 1 and 2).

Of the patients with Eastern Cooperative Oncology Group performance status (ECOG PS) data, most of those with ECOG PS of 0 were tested for biomarker(s), including from 63% (Italy) to 92% (Korea). Of those with ECOG PS of 1, from 50% (Germany and Brazil) to 89% (Taiwan) were tested.

Overall, of the patients who received a test, 63% and 33% received second- and third-line therapy, respectively; whereas of patients who were not tested 48% and 19% received second- and third-line therapy, respectively (Tables 1 and 2).

The majority of patients with nonsquamous NSCLC had one or more molecular tests (cumulative number, could be any test type), namely, from 54% in Brazil to 91% in Taiwan (Table 3). A median of 1 molecular test was performed for each patient with nonsquamous NSCLC from all countries except Germany (median, 2) and Korea (median, 3), with range of 1–2 or 1–3 tests in all countries except Spain (1–10), Germany (1–8), Australia (1–5), and Korea (1–5).

**Table 3:** Molecular test-related characteristics—at the test level—for nonsquamous aNSCLC in each country.

	Italy	Spain	Germany	Australia	Japan	Korea	Taiwan	Brazil
Characteristic	N = 121	N = 140	N = 108	N = 161	N = 129	N = 113	N = 201	N = 132
Patients tested, n (%)	79 (65)	119 (85)	71 (66)	115 (71)	110 (85)	101 (89)	183 (91)	71 (54)
Total number of molecular tests	111	201	167	179	133	248	189	86
No. molecular tests per patient, mean (SD)	1.4 (0.5)	1.7 (1.3)	2.4 (1.3)	1.6 (0.9)	1.2 (0.4)	2.5 (1.1)	1.0 (0.2)	1.2 (0.5)
Median (range)	1 (1–2)	1 (1–10)	2 (1–8)	1 (1–5)	1 (1–2)	3 (1–5)	1 (1–2)	1 (1–3)
Molecular test, n (% of tests)								
*ALK* rearrangement	30 (27)	39 (19)	39 (23)	26 (15)	25 (19)	55 (22)	5 (3)	14 (16)
*EGFR* mutation	76 (69)	113 (56)	68 (41)	114 (64)	107 (81)	108 (44)	184 (97)	70 (81)
KRAS	5 (5)	4 (2)	24 (14)	22 (12)	1 (1)	70 (28)	0	1 (1)
Other^a^	0	45 (22)	36 (22)	17 (10)	0	15 (6)	0	1 (1)
*EGFR* mutation status, n (% of EGFR tests)								
Positive	18 (24)	19 (17)	19 (28)	26 (23)	45 (42)	45 (42)	124 (67)	12 (17)
Negative	56 (74)	90 (80)	49 (72)	86 (75)	61 (57)	62 (57)	59 (32)	52 (74)
Unknown	2 (3)	4 (4)	0	2 (2)	1 (1)	1 (1)	1 (1)	6 (9)
*ALK* rearrangement status, n (% of ALK tests)								
Positive	1 (3)	2 (5)	2 (5)	4 (15)	2 (8)	9 (16)	3 (60)	0
Negative	28 (93)	36 (92)	36 (92)	20 (77)	23 (92)	43 (78)	1 (20)	12 (86)
Unknown	1 (3)	1 (3)	1 (3)	2 (8)	0	3 (6)	1 (20)	2 (14)
Type of tissue used^b^								
New, n (% of tests)	15 (14)	25 (12)	79 (48)	54 (30)	77 (58)	104 (42)	64 (34)	30 (35)
Archival, n (% of tests)	93 (86)	176 (88)	87 (52)	125 (70)	56 (42)	144 (58)	125 (66)	55 (65)
Timing of molecular tests, n (% of tests)^c^								
Before confirmed aNSCLC diagnosis	21 (19)	46 (23)	11 (7)	27 (15)	76 (57)	124 (50)	60 (32)	10 (12)
Before start of 1L, after confirmed diagnosis	60 (54)	127 (63)	94 (57)	99 (55)	46 (35)	92 (37)	108 (57)	53 (62)
Before start of 2L, after 1L therapy	20 (18)	19 (10)	43 (26)	45 (25)	4 (3)	15 (6)	15 (8)	15 (8)
Before start of 3L, after 2L therapy	7 (6)	4 (2)	9 (6)	7 (4)	4 (3)	5 (2)	2 (1)	2 (1)
After 3L	3 (3)	5 (3)	8 (5)	1 (1)	1 (1)	12 (5)	4 (2)	4 (2)
HC personnel ordering molecular test, n (% of tests)								
Oncologist	88 (90)	142 (89)	38 (25)	124 (75)	6 (5)	139 (57)	2 (1)	2 (1)
Pulmonologist	2 (2)	5 (3)	88 (59)	14 (8)	113 (93)	96 (39)	96 (62)	0
Pathologist	2 (2)	6 (4)	1 (1)	1 (1)	1 (1)	0	3 (2)	1 (1)
Thoracic surgeon	5 (5)	1 (1)	5 (3)	8 (5)	1 (1)	1 (0)	11 (7)	1 (1)
Other	0	6 (4)	18 (12)	19 (11)	1 (1)	10 (4)	43 (28)^d^	1 (1)
Missing/unknown test ordering data, n	13	41	17	13	11	2	34	9

1L, 2L, 3L, first-, second-, third-line; ALK, anaplastic lymphoma kinase; aNSCLC, advanced non-small cell lung cancer; EGFR, epidermal growth factor receptor; HC, health care.

^a^Types of “other” molecular tests were not defined.

^b^Tissue type data were missing for 3, 1, and 1 molecular tests in Italy, Germany, and Brazil, respectively.

^c^Molecular test timing data were missing for 2 tests each in Germany and Japan.

^d^In Taiwan, the treating physician ordered 40/155 tests (26%).

Molecular testing was conducted for 23% or fewer patients with squamous NSCLC except in Spain (42%) and Japan (40%; see Supporting S2 Table).

Testing for *EGFR* mutation was the most common molecular test in all countries and for both nonsquamous and squamous cohorts (Table 3 and Supporting S2 Table). In the nonsquamous cohorts, of all molecular tests conducted in each country, the percentages of EGFR tests ranged from 41% in Germany to 97% in Taiwan, while ALK tests comprised 3% (Taiwan) to 27% (Italy) of all molecular tests (Table 3). The percentages of positive *EGFR* mutation tests for nonsquamous NSCLC were 17% in Brazil and Spain, 23% in Australia, 24% in Italy, 28% in Germany, 42% in Japan and Korea, and 67% in Taiwan (Table 3). The percentages of positive *ALK* rearrangement tests for nonsquamous NSCLC were 0 in Brazil, 3% in Italy, 5% in Spain and Germany, 8% in Japan, 15% in Australia, 16% in Korea; only 5 *ALK* rearrangement tests were run in Taiwan, of which 3 (60%) were positive.

The majority of molecular tests (Table 3, Supporting S2 Table) and of the EGFR and ALK tests (Supporting S3 Table) were run before the initiation of first-line therapy. In Japan and Korea these tests were run most commonly before the confirmed NSCLC diagnosis and in the other countries most commonly after the confirmed diagnosis but before first-line therapy. A few tests for the nonsquamous cohorts in each country, but including 25–26% of those in Germany and Australia, were conducted after first-line therapy and before the start of second-line therapy (Table 3 and Supporting S2 and S3 Tables). Overall, 12% (Germany) or fewer molecular tests in each country were conducted after second-line therapy (Supporting S2 Table).

Both archival tissue and newly collected tissue were used for testing. Archival tissue was used most commonly in Italy (84%), Spain (83%), Australia (69%), Korea (58%), Taiwan (66%), and Brazil (66%; Table 3 and Supporting S2 Table). In Germany approximately half of molecular tests were run using new and half, archival tissue (49% and 51%, respectively), while in Japan, archival tissue was used more frequently for squamous NSCLC (57%) and new tissue for nonsquamous NSCLC (58%).

The median turn-around time for results of the *EGFR* test ranged from 8 (Japan) to 17 (Australia) days, depending on country, for patients with one test (Supporting S3 Table). For the *ALK* test, the corresponding range was a median of 3 (Italy) to 16 (Korea) days.

The health care provider who ordered the molecular tests was the oncologist most commonly in Italy (91%), Spain (86%), Australia (74%), Korea (57%), and Brazil (95%) and the pulmonologist most commonly in Germany (61%), Japan (92%), and Taiwan (61%; Table 3 and Supporting S2 Table).

### Biopsy patterns

Ninety percent or more of patients in each country, and up to 99% in Brazil, underwent a biopsy procedure, except in Spain (85%) and Taiwan (84%) (Supporting S4 Table). Over 94% of biopsies in each country were for diagnostic purposes, and the timing of the biopsy procedures was most commonly before the diagnosis, although in Taiwan 28% of biopsies were done after the NSCLC diagnosis and before the start of first-line therapy. The use of biopsies for molecular testing purposes ranged from 10% in Korea and Brazil to 30% in Taiwan and was most commonly done for nonsquamous NSCLC in all countries. The primary tumour was the most common biopsy site, and lymph nodes were the second most common site (Supporting S4 Table).

Several different biopsy methods were employed in each country, including transbronchial biopsy or brush biopsy, fine-needle biopsy, direct biopsy, surgical biopsy, and endobronchial/endoscopic-guided biopsy. Japan was the only country where over half of biopsies were performed with one technique, namely, 55% via transbronchial or brush biopsy (Supporting S4 Table). In the other countries, the most common methods of biopsy, used for 27% to 36% of biopsies, were transbronchial or brush biopsy (in Italy and Spain), fine needle biopsy (in Australia, Korea, and Taiwan), and direct biopsy (in Germany and Brazil).

Few patients in each country had a rebiopsy (4–9%), the exceptions being in Germany and Korea, where 20% had a rebiopsy, including 10 (36%) patients in the German squamous cohort and 26 (23%) in the Korean nonsquamous cohort. The rebiopsies were done most commonly for diagnostic purposes and secondarily for molecular testing (Supporting S4 Table).

### Treatment patterns by predictive biomarker testing status and results

Treatment patterns for first- through third-line therapy varied considerably according to receipt of a test (yes/no) and mutation status amongst those who received an EGFR-mutation or ALK-rearrangement test (*EGFR/ALK*-positive or *EGFR/ALK*-negative/unknown) (Tables 4 and 5; Supporting S5 Table).

**Table 4:** Treatment patterns by predictive biomarker testing (*EGFR* mutation and *ALK* rearrangement) and mutation status for patients with nonsquamous NSCLC in Italy, Spain, Germany, and Australia.

	Italy		Spain		Germany		Australia	
Patients who were tested^a^	EGFR/ALK+	EGFR/ALK –/unk	EGFR/ALK+	EGFR/ALK –/unk	EGFR/ALK+	EGFR/ALK –/unk	EGFR/ALK+	EGFR/ALK –/unk
**Systemic therapy**^**b**^	**N = 18**	**N = 60**	**N = 19**	**N = 93**	**N = 20**	**N = 50**	**N = 29**	**N = 86**
First-line therapy, n (%)	18 (100)	60 (100)	19 (100)	93 (100)	20 (100)	50 (100)	29 (100)	86 (100)
Platinum-based combination	7 (39)	58 (97)	10 (53)	87 (94)	13 (65)	47 (94)	15 (52)	79 (92)
Non-platinum combination	0	0	2 (11)	0	0	0	0	0
Single agents	0	2 (3)	0	5 (5)	1 (5)	3 (6)	0	7 (8)
EGFR/ALK TKI	11 (61)	0	7 (37)	1 (1)	6 (30)	0	14 (48)	0
Second-line therapy, n	N = 10	N = 39	N = 11	N = 47	N = 13	N = 22	N = 22	N = 58
Platinum-based combination	5 (50)	2 (5)	4 (36)	10 (21)	3 (23)	7 (32)	8 (36)	8 (14)
Non-platinum combination	0	0	2 (18)	2 (4)	1 (8)	2 (9)	0	0
Single agents	1 (10)	21 (54)	1 (9)	22 (47)	3 (23)	7 (32)	3 (14)	44 (76)
EGFR/ALK TKI	4 (40)	16 (41)	4 (36)	11 (23)	6 (46)	6 (27)	11 (50)	6 (10)
Other anti-NSCLC agent	0	0	0	2 (4)	0	0	0	0
Third-line therapy, n	N = 3	N = 21	N = 4	N = 26	N = 7	N = 10	N = 13	N = 20
Platinum-based combination	0	0	2 (50)	3 (12)	5 (71)	2 (20)	3 (23)	2 (10)
Non-platinum combination	0	0	1 (25)	4 (15)	0	2 (20)	2 (15)	0
Single agents	2 (67)	13 (62)	0	8 (31)	1 (14)	6 (60)	2 (15)	9 (45)
EGFR/ALK TKI	1 (33)	7 (33)	1 (25)	11 (42)	1 (14)	0	2 (15)	9 (45)
Other anti-NSCLC agent	0	1 (5)	0	0	0	0	4 (31)	0
**Patients who were not tested**^**a**^	**N = 43**		**N = 28**		**N = 38**		**N = 46**	
First-line therapy								
Platinum-based combination	36 (84)		25 (86)		31 (82)		37 (80)	
Non-platinum combination	0		1 (3)		0		0	
Single agents	6 (14)		2 (7)		7 (18)		9 (20)	
EGFR/ALK TKI	1 (2)		1 (4)		0		0	
Other anti-NSCLC agent	0		0		0		0	
Second-line therapy, n	N = 26		N = 12		N = 11		N = 20	
Platinum-based combination	2 (8)		4 (33)		3 (27)		3 (15)	
Non-platinum combination	0		0		2 (18)		0	
Single agents	15 (58)		7 (58)		5 (45)		13 (65)	
EGFR/ALK TKI	9 (35)		1 (8)		1 (9)		4 (20)	
Other anti-NSCLC agent	0		0		0		0	
Third-line therapy, n	N = 8		N = 5		N = 5		N = 7	
Platinum-based combination	1 (13)		1 (20)		0		1 (14)	
Non-platinum combination	0		1 (20)		0		1 (14)	
Single agents	5 (63)		2 (40)		3 (60)		3 (43)	
EGFR/ALK TKI	2 (25)		1 (20)		2 (40)		2 (29)	
Other anti-NSCLC agent	0		0		0		0	

ALK, anaplastic lymphoma kinase; EGFR, epidermal growth factor receptor; NSCLC, non-small cell lung cancer; TKI,tyrosine kinase inhibitor; unk unknown.

^a^Tested vs. not tested for *EGFR* mutation and/or *ALK* rearrangement.

^b^The five systemic therapy categories were defined as follows

• Platinum-based combination: regimen with two or more anticancer therapies including carboplatin or cisplatin.

• Non-platinum combination: regimen with two or more anticancer therapies not including carboplatin or cisplatin (can contain bevacizumab in combination with other non-platinum drug).

• Single agent: regimen of one anticancer drug that was not an EGFR or ALK tyrosine kinase inhibitor (TKI).

• EGFR/ALK TKI: monotherapy with anti-EGFR (erlotinib, gefitinib, afatinib) or anti-ALK agent (crizotinib, ceritinib).

• Other NSCLC anticancer agent: any other agent not included in the prior categories, eg, TS-1 (oral anticancer drug composed of tegafur, gimestat, and otastat potassium at a molar ratio of 1:0.4:1).

**Table 5:** Treatment patterns by predictive biomarker testing (*EGFR* mutation and *ALK* rearrangement) and mutation status for patients with nonsquamous NSCLC in Japan, Korea, Taiwan, and Brazil.

	Japan		Korea		Taiwan		Brazil	
Patients who were tested^a^	EGFR/ALK+	EGFR/ALK –/unk	EGFR/ALK+	EGFR/ALK –/unk	EGFR/ALK+	EGFR/ALK –/unk	EGFR/ALK+	EGFR/ALK –/unk
**Systemic therapy**^**b**^	**N = 46**	**N = 64**	**N = 48**	**N = 53**	**N = 126**	**N = 57**	**N = 12**	**N = 58**
First-line therapy, n (%)	46 (100)	64 (100)	48 (100)	53 (100)	126 (100)	57 (100)	12 (100)	58 (100)
Platinum-based combination	4 (9)	56 (88)	24 (50)	48 (91)	9 (7)	20 (35)	8 (67)	57 (98)
Non-platinum combination	0	0	0	1 (2)	15 (12)	8 (14)	0	0
Single agents	1 (2)	6 (9)	2 (4)	2 (4)	1 (1)	23 (40)	0	1 (2)
EGFR/ALK TKI	41 (89)	0	22 (46)	2 (4)	101 (80)	6 (11)	4 (33)	0
Other anti-NSCLC agent	0	2 (3)	0	0	0	0	0	0
Second-line therapy, n	N = 30	N = 33	N = 34	N = 32	N = 85	N = 42	N = 7	N = 41
Platinum-based combination	13 (43)	5 (15)	9 (27)	1 (3)	40 (47)	3 (7)	2 (29)	9 (22)
Non-platinum combination	0	1 (3)	0	0	8 (9)	10 (24)	0	1 (2)
Single agents	2 (7)	24 (73)	9 (27)	13 (41)	29 (34)	14 (33)	4 (57)	27 (66)
EGFR/ALK TKI	15 (50)	3 (9)	16 (47)	18 (56)	8 (9)	15 (36)	1 (14)	4 (10)
Third-line therapy, n	N = 15	N = 16	N = 18	N = 22	N = 54	N = 23	N = 3	N = 17
Platinum-based combination	3 (20)	6 (38)	0	2 (9)	2 (4)	0	0	3 (18)
Non-platinum combination	2 (13)	0	1 (6)	1 (5)	4 (7)	5 (22)	1 (33)	2 (12)
Single agents	1 (7)	7 (44)	12 (67)	13 (59)	14 (26)	9 (39)	1 (33)	7 (41)
EGFR/ALK TKI	9 (60)	3 (19)	5 (28)	6 (27)	34 (63)	8 (35)	1 (33)	5 (29)
Other anti-NSCLC agent	0	0	0	0	0	1 (4)	0	0
**Patients who were not tested**^**a**^	**N = 19**		**N = 12**		**N = 18**		**N = 62**	
First-line therapy								
Platinum-based combination	18 (95)		11 (92)		4 (22)		56 (90)	
Non-platinum combination	0		0		2 (11)		0	
Single agents	1 (5)		1 (8)		8 (44)		6 (10)	
EGFR/ALK TKI	0		0		4 (22)		0	
Second-line therapy, n	N = 10		N = 8		N = 15		N = 23	
Platinum-based combination	4 (40)		0		1 (7)		2 (9)	
Non-platinum combination	1 (10)		0		9 (60)		0	
Single agents	5 (50)		2 (25)		1 (7)		20 (87)	
EGFR/ALK TKI	0		6 (75)		4 (27)		1 (4)	
Third-line therapy, n	N = 5		N = 6		N = 7		N = 2	
Platinum-based combination	1 (20)		1 (17)		0		0	
Non-platinum combination	1 (20		1 (17)		4 (57)		0	
Single agents	3 (60)		3 (50)		1 (14)		1 (50)	
EGFR/ALK TKI	0		1 (17)		2 (29)		1 (50)	

ALK, anaplastic lymphoma kinase; EGFR, epidermal growth factor receptor; NSCLC, non-small cell lung cancer; TKI, tyrosine kinase inhibitor; unk, unknown.

^a^Tested vs. not tested for *EGFR* mutation and/or *ALK* rearrangement.

^b^The five systemic therapy categories were defined as follows

• Platinum-based combination: regimen with two or more anticancer therapies including carboplatin or cisplatin.

• Non-platinum combination: regimen with two or more anticancer therapies not including carboplatin or cisplatin (can contain bevacizumab in combination with other non-platinum drug).

• Single agent: regimen of one anticancer drug that was not an EGFR or ALK tyrosine kinase inhibitor (TKI).

• EGFR/ALK TKI: monotherapy with anti-EGFR (erlotinib, gefitinib, afatinib) or anti-ALK agent (crizotinib, ceritinib).

• Other NSCLC anticancer agent: any other agent not included in the prior categories, eg, TS-1 (oral anticancer drug composed of tegafur, gimestat, and otastat potassium at a molar ratio of 1:0.4:1).

For nonsquamous NSCLC (Tables 4 and 5), the percentages of patients with *EGFR/ALK*-positive status who received a first-line EGFR/ALK TKI ranged from 30% in Germany to 89% in Japan. The corresponding percentages in second-line were 9% (Taiwan) to 50% Australia and Japan, and in third-line were 14% (Germany) to 63% (Taiwan). Instead, first-line platinum-based combinations were administered to 88–98% of patients with nonsquamous NSCLC who had negative/inconclusive test results in each country, and to 80–95% of those who were not tested in all countries except Taiwan (Tables 4 and 5). The first-line regimens were more varied in Taiwan, where patients with negative/inconclusive test results received platinum-based combinations and single agents in roughly equal proportions (35% and 40%, respectively), and a minority received non-platinum combinations (14%) and EGFR/ALK TKIs (11%). The 18 patients in Taiwan who were not tested received single agents (44%) most commonly, followed by platinum-based combinations (22%), EGFR/ALK TKIs (22%), and non-platinum combinations (11%; Table 5).

Among patients with squamous NSCLC, there were few with positive *EGFR/ALK* status (1 each in Germany, Japan, and Korea and 2 in Australia); most of these patients with *EGFR/ALK*-positive tumors received an EGFR/ALK TKI in first- or second-line (Supporting S5 Table).

### Overall survival by predictive biomarker testing status and results

**Fig 1:**
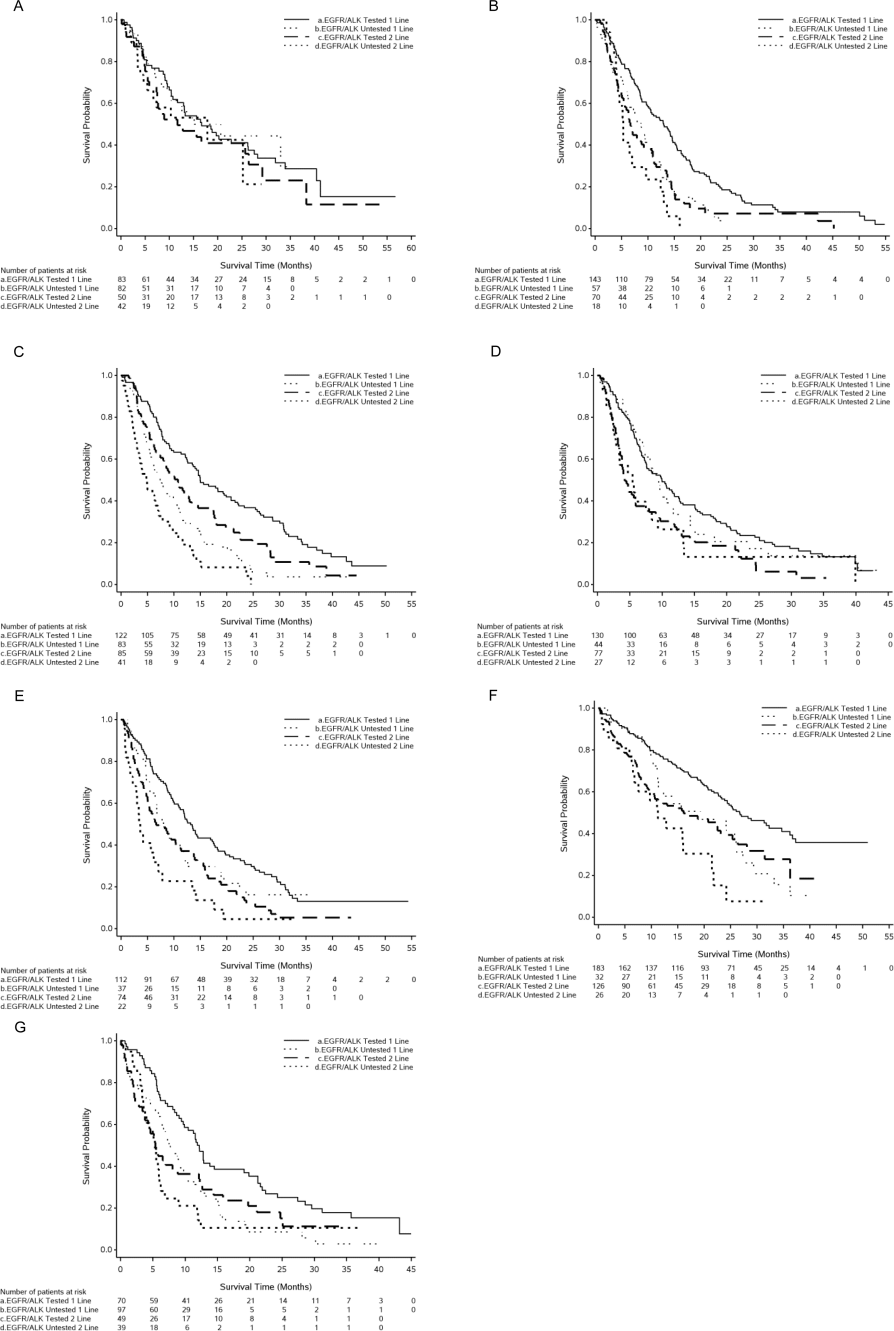
**Kaplan-Meier plots of overall survival from initiation of first- and second-line therapy by predictive biomarker testing status (*EGFR* mutation and/or *ALK* rearrangement—tested / untested) for each country:** (A) Italy, (B) Spain, (C) Australia, (D) Japan, (E) Korea, (F) Taiwan, (G) Brazil.

For patients who had an *EGFR* or *ALK* test, irrespective of NSCLC histology, the median OS (95% CI) from initiation of first-line therapy ranged in months from 10.0 (7.4–12.6) in Japan to 26.7 (22.7–34.7) in Taiwan. For those who were not tested, the median OS ranged from 7.6 months in Australia (95% CI, 5.8–10.3) and Brazil (95% CI, 6.3–9.3) to 19.3 (11.1–27.3) in Taiwan. Of the nonsquamous cohorts, the shortest median OS was recorded for those not tested in Korea (5.7 months) and the longest was for those tested in Italy (40.4 months; further details in Table 6). Survival is depicted graphically by predictive biomarker testing status (tested / not tested) for each country in Fig 1.

**Table 6:** Overall survival by NSCLC histology, predictive biomarker testing, and mutation status (EGFR mutation and/or ALK rearrangement).

	Italy	Spain	Australia	Korea	Taiwan	Japan	Brazil
Characteristic	N = 174	N = 202	N = 208	N = 150	N = 217	N = 175	N = 175
**Patients who were tested, n**	89	154	126	114	185	130	75
**Median OS (95% CI) start of 1L, months**							
Squamous	1.9 (NA)	13.4 (1.7–23.7)	8.3 (0.2–13.7)	13.0 (8.4–NA)	7.0 (6.9–7.0)	12.7 (6.5–22.0)	20.7 (5.7–35.7)
Nonsquamous, EGFR/ALK+	40.4 (4.7–NA)	15.1 (8.7–20.7)	34.3 (26.2–43.7)	14.5 (11.0–23.3)	28.3 (23.3–37.3)	17.9 (9.9–24.4)	11.9 (2.6–22.4)
Nonsquamous, EGFR/ALK-/unk	18.6 (10.4–26.3)	13.0 (8.6–15.3)	12.6 (7.9–17.9)	11.8 (6.9–17.6)	25.1 (10.9–36.7)	6.9 (5.6–10.0)	12.0 (9.4–14.5)
All patients	16.7 (10.7–26.3)	13.0 (10.0–14.7)	15.0 (12.4–20.8)	13.2 (10.1–17.5)	26.7 (22.7–34.7)	10.0 (7.4–12.6)	12.0 (9.4–14.5)
**Median OS (95% CI) start of 2L, months**							
Squamous	NA	14.2 (1.9–42.2)	3.4 (NA)	5.0 (1.0–5.8)	3.8 (2.9–4.8)	8.0 (3.1–15.1)	NA
Nonsquamous, EGFR/ALK+	26.4 (0.9–NA)	6.8 (1.9–14.4)	17.5 (8.2–35.6)	10.7 (4.8–16.5)	15.8 (9.6–26.4)	4.0 (3.3–12.0)	4.2 (0.1–14.4)
Nonsquamous, EGFR/ALK-/unk	11.7 (7.4–29.2)	6.3 (4.4–10.7)	8.9 (5.8–12.0)	6.2 (3.8–11.4)	18.8 (7.4–31.5)	4.0 (2.8–4.8)	5.8 (3.4–12.3)
All patients	11.7 (7.4–26.4)	6.7 (5.4–10.7)	10.2 (6.8–13.0)	6.7 (5.0–10.7)	16.1 (9.6–23.1)	4.2 (3.5–5.8)	5.4 (3.6–8.8)
**Patients who were not tested, n**	85	48	82	36	32	45	100
**Median OS (95% CI) start of 1L, months**							
Squamous	14.8 (7.0–NA)	10.3 (6.1–13.5)	6.1 (4.3–12.2)	9.8 (6.8–17.8)	15.7 (2.1–26.1)	9.7 (5.9–11.7)	10.5 (6.9–19.5)
Nonsquamous	33.0 (10.4–NA)	8.3 (3.5–11.7)	9.2 (5.9–11.6)	5.7 (1.8–22.4)	19.3 (11.2–33.3)	8.5 (3.9–26.4)	6.9 (5.4–9.2)
All patients	15.1 (10.3–NA)	8.7 (6.1–10.7)	7.6 (5.8–10.3)	8.1 (5.5–12.1)	19.3 (11.1–27.3)	9.5 (7.0–11.7)	7.6 (6.3–9.3)
**Median OS (95% CI) start of 2L, months**							
Squamous	NA (4.7–NA)	3.8 (2.2–13.6)	3.6 (0.9–6.0)	3.2 (1.5–6.2)	6.8 (1.9–NA)	4.7 (2.0–8.4)	6.2 (1.9–11.9)
Nonsquamous	17.9 (3.4–NA)	6.5 (4.6–12.4)	7.2 (3.5–10.8)	5.6 (0.7–17.6)	15.9 (6.3–21.8)	7.5 (0.8–39.9)	5.1 (3.5–6.0)
All patients	17.9 (5.5–NA)	5.3 (3.9–9.7)	4.9 (3.0–7.2)	3.5 (1.7–7.1)	11.2 (6.5–21.5)	5.4 (2.6–8.4)	5.3 (3.6–6.0)

1L, first-line therapy; 2L, second-line therapy; ALK, anaplastic lymphoma kinase; EGFR, epidermal growth factor receptor; NA, not available (median not reached or sub-cohort sample size too small); NSCLC, non-small cell lung cancer; OS, overall survival; unk unknown.

## Discussion

This large observational study enabled us to examine and describe the frequency of molecular testing for advanced NSCLC by histologic type, the characteristics of patients who were tested, and treatments received and survival patterns in relation to receipt of a test and mutation status in eight countries. We found high proportions of patients with nonsquamous histology receiving at least one molecular test during the study period, namely, 54% (Brazil) to 91% (Taiwan) of patients, including 85% or more in Spain and the three Asian countries. A numerically higher proportion of patients with nonsquamous (vs. squamous) NSCLC, female (vs. male) patients, Asian (vs. other ethnicity) patients, never smokers and ex-smokers (vs. current smokers), and patients with stage IV (vs. stage IIIB) NSCLC received a molecular test. Testing for *EGFR* mutation was the most common molecular test in all countries, and the overall percentages of positive tests for *EGFR* mutation in nonsquamous NSCLC were 17–28% in the non-Asian countries and 42–67% in the Asian countries. Treatment patterns overall corresponded with molecular testing status: more patients with *EGFR/ALK*-positive status (vs. negative/unknown status) received targeted therapy, especially for first-line therapy, while most patients with negative/inconclusive test results or who were not tested received platinum-based combinations as first-line therapy in all countries except Taiwan, where regimens were more varied. The median OS from start of first-line therapy ranged from 10.0 (Japan) to 26.7 (Taiwan) months for patients who were tested and 7.6 (Australia/Brazil) to 19.3 (Taiwan) months for those not tested.

This study is descriptive, and we cannot directly compare our findings amongst countries because of differences in the health care environments as well as differences in approval and reimbursement timelines. Nonetheless, some general comments can be made.

The frequency of molecular testing differed across countries, although *EGFR* mutation testing was the most common in all. We found that the rate of *EGFR* mutation testing was higher in Asian as compared with European countries, except Spain. The testing rate was lowest overall in Brazil, where *EGFR* mutation and *ALK* rearrangement testing, as well as ALK inhibitor therapy, were not covered in the public health care system during the study period. Moreover, according to one of the authors (CHB), the number of testing facilities is limited in Brazil, presenting a further challenge to routine molecular testing. Testing is always associated with potential access to the associated drug. This fact was evident in Taiwan, where EGFR tests represented 97% and ALK tests only 3% of tests conducted for nonsquamous NSCLC. We speculate this was because drug reimbursement in Taiwan is based on approved indication(s), and, while gefitinib was available, the first ALK inhibitor to be approved in Taiwan (crizotinib) was not approved until after our study (September 2015), when it was approved for second-line treatment of NSCLC positive for *ALK* rearrangement.

As we expected, testing was more common for nonsquamous NSCLC, with a median of 1 test performed per patient with nonsquamous NSCLC except in Korea and Germany (median of 2 and 3 tests, respectively). Tests were submitted most commonly by the oncologists and/or pulmonologists, depending on country. Thus our findings suggest that the rate of reflex testing was low in all countries because few tests were ordered by pathologists. Reflex testing for predictive biomarkers at the time of diagnosis by the pathologist can improve the proportion of patients tested and reduce time to treatment decisions [20, 36, 37]. Nonetheless, in Korea and Japan, the first test occurred often before a confirmed diagnosis (50% and 57% of tests, respectively), suggesting that patients were suspected to have nonsquamous histology based on patient characteristics and clinical symptoms, and molecular testing was ordered along with histology diagnosis to maximize efficiency. In the other countries, over 50% of molecular tests were conducted after the confirmed diagnosis and before the start of first-line therapy. For molecular testing purposes, archival tissue, defined as tissue collected at first biopsy and preserved for future testing purposes, was used most commonly.

Positive tests for *EGFR* mutation were more common in the Asian countries (Japan, Korea, and Taiwan) than in Brazil, Australia, and the European countries, as would be expected [5, 14]. The percentage of EGFR tests that were positive was especially high in Taiwan for nonsquamous NSCLC (67%), perhaps because of the relatively high percentages of women and never/ex-smokers. A positive ALK rearrangement test for nonsquamous NSCLC varied from 0 in Brazil, to 3–5% in European countries, and 8–16% in Asian countries and Australia. In Taiwan, 60% were positive, but only five ALK tests were run, too few from which to draw conclusions.

Different from molecular testing rates, the biopsy rates were similar across countries, with on average 1 biopsy per patient. Our data suggest that biopsies were performed mostly to determine histology and in certain countries also for biomarker testing, specifically in the three European countries and Taiwan, where one-quarter or more of biopsies were used for biomarker testing. The majority of biopsies occurred before the start of first-line therapy, which is as expected. We found that rates of rebiopsy were low, and rebiopsies were done most commonly for diagnostic purposes, and sometimes for molecular testing.

Our findings are purely descriptive, hence we cannot make conclusive comparative statements across countries or within a country, and the findings reflect aggregate data; therefore, we cannot show a causal relationship. Nonetheless, we noted that a higher percentage of patients who were tested, particularly those with tumors positive for *EGFR* mutation or *ALK* rearrangement, received targeted therapy, while a higher percentage of those who were not tested received platinum-based therapies or single agents. Moreover, we noted survival differences between cohorts who were tested and those who never received a molecular test. These differences could be due to inherent patient characteristics or the fact that the majority of patients in the tested cohorts received a targeted therapy sometime during their course of treatment. Clinical trials have shown that targeted therapies have improved survival compared with traditional chemotherapy [5]. Our study was not powered to show this causal relationship, and further real-world studies are needed to explore the effectiveness of testing and receipt of targeted therapy for advanced NSCLC. While testing appeared to have had an impact on survival, we recognize that in many countries a substantial proportion of patients still remained untested.

Strengths of this study include the large numbers of patients and the recent data (2011–2016) detailing molecular testing and biopsy patterns in eight countries by NSCLC histology (squamous versus nonsquamous NSCLC). Our findings provide an update to the results of prior multinational, observational studies [27, 29, 38], illustrating the rapidly changing landscape of NSCLC therapy. The importance of real-world data is increasingly recognized as a means to understand routine clinical practice with associated outcomes, information that can be used to improve care [31, 39]. These real-world findings provide data to benchmark and establish the standard of care before the newer immunotherapy treatments came into use.

Several limitations of this retrospective observational study need mention. We worked with a convenience sample of study sites that routinely manage patients with NSCLC; therefore, treatment practices and clinical assessments were not standardized, reviewed by a single panel, or assessed as to whether they represented country-wide practices. We did not assess the quality of the testing facilities. Nor did we assess the methods used for molecular testing, an area of interest as new testing methods are becoming available; in addition, the testing method could have influenced test turnaround times. Patients were selected for study consecutively, working backward in time; nonetheless, selection bias is possible, particularly because the number of centers and of patients in each country were relatively modest. Moreover, we did not determine treatment regimens sequentially according to molecular test results. Finally, the testing and treatment alternatives for NSCLC have already increased since our study; however, our findings provide benchmark data regarding the standard of care in eight countries before the use of newer immunotherapy treatments.

Many unanswered questions and issues remain. Barriers to testing need to be identified—including the roles of reimbursement criteria, availability of treatment, and access to education for physicians—and practical strategies to address these barriers need to be developed. Strategies will necessarily be context and country dependent but could include physician education, development of regional certified laboratories, industry-sponsored programs for testing, and resolving reimbursement issues. The use of pembrolizumab as first-line therapy is indicated for patients with advanced NSCLC tumors positive for PD-L1 and *EGFR/ALK* wild-type, emphasizing the need for molecular testing before therapy. Our results suggest a need to improve *ALK* rearrangement testing rates in particular. Moreover, new biomarkers are being discovered, such as ROS1.

The treatment landscape for NSCLC is rapidly changing with improved tumor characterization and ongoing development of targeted therapies and immunotherapies. This study provides descriptive information that can be used to understand testing and treatment patterns to help identify current clinical needs. While a large proportion of patients are not tested, our findings suggest that a majority of patients are being tested for predictive biomarkers and are receiving treatment in accordance with their mutation status.

## Supporting information

S1 File Supplemental methods. (DOCX)

S1 Table Listing of PIvOTAL study ethics committee names and locations. (XLSX)

S2 Table Molecular test-related characteristics—at the test level—in all 8 countries, by NSCLC histology. (XLSX)

S3 Table Number, results, and timing of tests for EGFR mutation and ALK rearrangement, at the test level overall in each country. (XLSX)

S4 Table Biopsy-related characteristics for all 8 countries. (XLSX)

S5 Table Treatment patterns by predictive biomarker testing (EGFR mutation and ALK rearrangement) and mutation status, by NSCLC histology. (XLSX)

## References

[1] World Health Organization. Fact Sheets: Cancer. February 2018. Available from: http://www.who.int/en/news-room/fact-sheets/detail/cancer.

[2] International Agency for Research on Cancer. Cancer Today. Available from: http://gco.iarc.fr/today.

[3] Cheng TY, Cramb SM, Baade PD, Youlden DR, Nwogu C, Reid ME. The international epidemiology of lung cancer: latest trends, disparities, and tumor characteristics. J Thorac Oncol. 2016;11:1653–71. 10.1016/j.jtho.2016.05.021 27364315 PMC5512876

[4] American Cancer Society. About non-small cell lung cancer. Available from: https://www.cancer.org/cancer/non-small-cell-lung-cancer/about.html.

[5] NCCN Guidelines: Non-small cell lung cancer. Version 4.2017. Available from: http://www.nccn.org/professionals/physician_gls/f_guidelines.asp.

[6] Tanner NT, Pastis NJ, Sherman C, Simon GR, Lewin D, Silvestri GA. The role of molecular analyses in the era of personalized therapy for advanced NSCLC. Lung Cancer. 2012;76:131–7. 10.1016/j.lungcan.2011.11.013 22176813 PMC3403712

[7] Travis WD, Brambilla E, Nicholson AG, Yatabe Y, Austin JH, Beasley MB, et al The 2015 World Health Organization classification of lung tumors: impact of genetic, clinical and radiologic advances since the 2004 classification. J Thorac Oncol. 2015;10:1243–60. 10.1097/JTO.0000000000000630 26291008

[8] Novello S, Barlesi F, Califano R, Cufer T, Ekman S, Levra MG, et al Metastatic non-small-cell lung cancer: ESMO Clinical Practice Guidelines for diagnosis, treatment and follow-up. Ann Oncol. 2016;27:v1–v27. 10.1093/annonc/mdw326 27664245

[9] Japan Lung Cancer Society. Treatment guidelines for lung cancer. Available from: http://www.haigan.gr.jp/guideline/2015/2/150002050100.html.

[10] Greenhalgh J, Dwan K, Boland A, Bates V, Vecchio F, Dundar Y, et al First-line treatment of advanced epidermal growth factor receptor (EGFR) mutation positive non-squamous non-small cell lung cancer. Cochrane Database Syst Rev. 2016:CD010383 10.1002/14651858.CD010383.pub2 27223332

[11] Solomon B. First-line treatment options for ALK-rearranged lung cancer. Lancet. 2017;389:884–6. 10.1016/S0140-6736(17)30124-1 28126330

[12] Gainor JF, Varghese AM, Ou SH, Kabraji S, Awad MM, Katayama R, et al ALK rearrangements are mutually exclusive with mutations in EGFR or KRAS: an analysis of 1,683 patients with non-small cell lung cancer. Clin Cancer Res. 2013;19:4273–81. 10.1158/1078-0432.CCR-13-0318 23729361 PMC3874127

[13] Kosaka T, Yatabe Y, Endoh H, Kuwano H, Takahashi T, Mitsudomi T. Mutations of the epidermal growth factor receptor gene in lung cancer: biological and clinical implications. Cancer Res. 2004;64:8919–23. 10.1158/0008-5472.CAN-04-2818 15604253

[14] Yatabe Y, Kerr KM, Utomo A, Rajadurai P, Tran VK, Du X, et al EGFR mutation testing practices within the Asia Pacific region: results of a multicenter diagnostic survey. J Thorac Oncol. 2015;10:438–45. 10.1097/JTO.0000000000000422 25376513 PMC4342317

[15] Shaw AT, Yeap BY, Mino-Kenudson M, Digumarthy SR, Costa DB, Heist RS, et al Clinical features and outcome of patients with non-small-cell lung cancer who harbor EML4-ALK. J Clin Oncol. 2009;27:4247–53. 10.1200/JCO.2009.22.6993 19667264 PMC2744268

[16] Sacher AG, Dahlberg SE, Heng J, Mach S, Janne PA, Oxnard GR. Association between younger age and targetable genomic alterations and prognosis in non-small-cell lung cancer. JAMA Oncol. 2016;2:313–20. 10.1001/jamaoncol.2015.4482 26720421 PMC4819418

[17] Leighl NB, Rekhtman N, Biermann WA, Huang J, Mino-Kenudson M, Ramalingam SS, et al Molecular testing for selection of patients with lung cancer for epidermal growth factor receptor and anaplastic lymphoma kinase tyrosine kinase inhibitors: American Society of Clinical Oncology endorsement of the College of American Pathologists/International Association for the Study of Lung Cancer/Association for Molecular Pathology Guideline. J Clin Oncol. 2014;32:3673–9. 10.1200/JCO.2014.57.3055 25311215 PMC5321089

[18] Salto-Tellez M, Tsao MS, Shih JY, Thongprasert S, Lu S, Chang GC, et al Clinical and testing protocols for the analysis of epidermal growth factor receptor mutations in East Asian patients with non-small cell lung cancer: a combined clinical-molecular pathological approach. J Thorac Oncol. 2011;6:1663–9. 10.1097/JTO.0b013e318227816a 21869714

[19] Kim H, Shim HS, Kim L, Kim TJ, Kwon KY, Lee GK, et al Guideline Recommendations for Testing of ALK Gene Rearrangement in Lung Cancer: A Proposal of the Korean Cardiopulmonary Pathology Study Group. Korean J Pathol. 2014;48:1–9. 10.4132/KoreanJPathol.2014.48.1.1 24627688 PMC3950228

[20] Cheema PK, Raphael S, El-Maraghi R, Li J, McClure R, Zibdawi L, et al Rate of EGFR mutation testing for patients with nonsquamous non-small-cell lung cancer with implementation of reflex testing by pathologists. Curr Oncol. 2017;24:16–22. 10.3747/co.24.3266 28270720 PMC5330624

[21] Reck M, Hermes A, Tan EH, Felip E, Klughammer B, Baselga J. Tissue sampling in lung cancer: a review in light of the MERIT experience. Lung Cancer. 2011;74:1–6. 10.1016/j.lungcan.2011.05.002 21658788

[22] Kerr KM. Personalized medicine for lung cancer: new challenges for pathology. Histopathology. 2012;60:531–46. 10.1111/j.1365-2559.2011.03854.x 21916947

[23] Hiley CT, Le Quesne J, Santis G, Sharpe R, de Castro DG, Middleton G, et al Challenges in molecular testing in non-small-cell lung cancer patients with advanced disease. Lancet. 2016;388:1002–11. 10.1016/S0140-6736(16)31340-X 27598680

[24] Thunnissen E, Kerr KM, Herth FJ, Lantuejoul S, Papotti M, Rintoul RC, et al The challenge of NSCLC diagnosis and predictive analysis on small samples. Practical approach of a working group. Lung Cancer. 2012;76:1–18. 10.1016/j.lungcan.2011.10.017 22138001

[25] Moro-Sibilot D, Vergnenegre A, Smit EF, Toy E, Parente B, Schmitz S, et al Second-line therapy for NSCLC in clinical practice: baseline results of the European SELECTTION observational study. Curr Med Res Opin. 2010;26:2661–72. 10.1185/03007995.2010.525489 20942749

[26] Vergnenegre A, Smit EF, Toy E, Parente B, Schmitz S, Kraaij K, et al Second-line therapy for non-small cell lung cancer in clinical practice: final results and treatment pathways from the SELECTTION observational study. Curr Med Res Opin. 2012;28:1253–62. 10.1185/03007995.2012.703133 22697276

[27] Schnabel PA, Smit E, Carpeno Jde C, Lesniewski-Kmak K, Aerts J, Kraaij K, et al Influence of histology and biomarkers on first-line treatment of advanced non-small cell lung cancer in routine care setting: baseline results of an observational study (FRAME). Lung Cancer. 2012;78:263–9. 10.1016/j.lungcan.2012.09.001 23040326

[28] Moro-Sibilot D, Smit E, de Castro Carpeno J, Lesniewski-Kmak K, Aerts J, Villatoro R, et al Outcomes and resource use of non-small cell lung cancer (NSCLC) patients treated with first-line platinum-based chemotherapy across Europe: FRAME prospective observational study. Lung Cancer. 2015;88:215–22. 10.1016/j.lungcan.2015.02.011 25748103

[29] Carrato A, Vergnenegre A, Thomas M, McBride K, Medina J, Cruciani G. Clinical management patterns and treatment outcomes in patients with non-small cell lung cancer (NSCLC) across Europe: EPICLIN-Lung study. Curr Med Res Opin. 2014;30:447–61. 10.1185/03007995.2013.860372 24168104

[30] Gridelli C, de Marinis F, Ardizzoni A, Novello S, Fontanini G, Cappuzzo F, et al Advanced non-small cell lung cancer management in patients progressing after first-line treatment: results of the cross-sectional phase of the Italian LIFE observational study. J Cancer Res Clin Oncol. 2014;140:1783–93. 10.1007/s00432-014-1715-2 24903964 PMC11823904

[31] von Verschuer U, Schnell R, Tessen HW, Eggert J, Binninger A, Spring L, et al Treatment, outcome and quality of life of 1239 patients with advanced non-small cell lung cancer—final results from the prospective German TLK cohort study. Lung Cancer. 2017;112:216–24. 10.1016/j.lungcan.2017.07.031 28916198

[32] De Geer A, Eriksson J, Finnern HW. A cross-country review of data collected on non-small cell lung cancer (NSCLC) patients in cancer registries, databases, retrospective and non-randomized prospective studies. J Med Econ. 2013;16:134–49. 10.3111/13696998.2012.703631 22702446

[33] de Castro J, Tagliaferri P, de Lima VCC, Ng S, Thomas M, Arunachalam A, et al Systemic therapy treatment patterns in patients with advanced non-small cell lung cancer (NSCLC): PIvOTAL study. Eur J Cancer Care (Engl). 2017;26:e12734 10.1111/ecc.12734 28748556 PMC5697695

[34] Isobe H, Mori K, Minato K, Katsura H, Taniguchi K, Arunachalam A, et al Real-world practice patterns for patients with advanced non-small cell lung cancer: multicenter retrospective cohort study in Japan. Lung Cancer (Auckl). 2017;8:191–206. 10.2147/LCTT.S140491 29123433 PMC5661576

[35] Lee DH, Isobe H, Wirtz H, Aleixo SB, Parente P, de Marinis F, et al Health care resource use among patients with advanced non-small cell lung cancer: the PIvOTAL retrospective observational study. BMC Health Serv Res. 2018;18:147 10.1186/s12913-018-2946-8 29490654 PMC5831211

[36] Lim C, Tsao MS, Le LW, Shepherd FA, Feld R, Burkes RL, et al Biomarker testing and time to treatment decision in patients with advanced nonsmall-cell lung cancer. Ann Oncol. 2015;26:1415–21. 10.1093/annonc/mdv208 25922063

[37] Jain A, Lim C, Gan EM, Ng DZ, Ng QS, Ang MK, et al Impact of smoking and brain metastasis on outcomes of advanced EGFR mutation lung adenocarcinoma patients treated with first line epidermal growth factor receptor tyrosine kinase inhibitors. PLoS One. 2015;10:e0123587 10.1371/journal.pone.0123587 25955322 PMC4425557

[38] Walters S, Maringe C, Coleman MP, Peake MD, Butler J, Young N, et al Lung cancer survival and stage at diagnosis in Australia, Canada, Denmark, Norway, Sweden and the UK: a population-based study, 2004–2007. Thorax. 2013;68:551–64. 10.1136/thoraxjnl-2012-202297 23399908

[39] Sherman RE, Anderson SA, Dal Pan GJ, Gray GW, Gross T, Hunter NL, et al Real-world evidence—what is it and what can it tell us? N Engl J Med. 2016;375:2293–7. 10.1056/NEJMsb1609216 27959688

