# Supplementary material for: Molecular testing and treatment patterns for patients with advanced non-small cell lung cancer: PIvOTAL observational study
Source: PLoS One. 2018 Aug 27;13(8):e0202865. doi: 10.1371/journal.pone.0202865 (PMC6110501; doi:10.1371/journal.pone.0202865)
Supplement: S1 File — (DOCX) [file pone.0202865.s001.docx]

**S1 File. Supplemental methods**

The eligibility period in Germany was extended to July 1, 2014, to recruit sufficient patient numbers.

Investigators were provided with guidelines for lung cancer staging according to the TNM classification of the Union for International Cancer Control [1] and the American Joint Committee on Cancer [2].

**Definitions**

Multiple predictive biomarker tests for *EGFR* mutation or *ALK* rearrangement could be conducted at the same or different time points, and the same tests could be repeated multiple times. The first biomarker test was defined as all tests performed on the first date of any biomarker test. So if a patient had multiple biomarker tests, all were considered the first biomarker test. The second biomarker test was defined as all tests on the second date of any biomarker test, with a similar definition for the third biomarker test. If multiple tests for a given biomarker test (*EGFR* mutation or *ALK* rearrangement) had conflicting results (positive vs. negative), we determined the patient’s test status based on treatment timing and clinical judgment.

The turnaround time for biomarker testing was defined as the difference in the number of working days from the date the clinician ordered the test until the date on which the clinician received the test results.

The earliest date of tissue sampling (biopsy) in the study period for an individual patient was considered the first biopsy. For the purposes of this study, any biopsy or tissue sampling performed after the first biopsy was considered a rebiopsy. Thus, a rebiopsy was a second biopsy procedure, which could potentially happen on the same day as the first biopsy. For patients with multiple biopsies on the first day, we randomly assigned the order to differentiate the first, second, and/or third biopsy when we populated the table of biopsy timing and clinical characteristics of rebiopsy.

New tissue was defined as tissue collected at the first and/or subsequent biopsy and used for testing within 60 days. Archival tissue was defined as tissue collected at first biopsy and preserved for future testing purposes.

Systemic therapies for NSCLC were categorized into five regimen types: platinum-based combinations (any drug in combination with cisplatin or carboplatin), non-platinum combinations, single agents, EGFR/ALK TKIs, and other regimen types.

**References**

1. American Joint Committee on Cancer, AJCC Lung Cancer Staging, 7th ed. 2010. Available from: https://cancerstaging.org/references-tools/deskreferences/Pages/default.aspx.

2. Union for International Cancer Control (UICC). TNM classification of malignant tumours, 7th ed. Geneva; 2009.
